# BCLAF1 Regulates Osteoarthritic Cartilage Degradation Through Interaction with LAMTOR2

**DOI:** 10.7150/ijbs.100396

**Published:** 2025-02-03

**Authors:** Song Li, Danni Luo, Yulin Liang, Yi Zou, Hongxu Pu, Meng Zheng, Yuting Wang, Xuying Sun, Hao Zhu, Yuanli Zhu, Liming Zhao, Jun Xiao

**Affiliations:** 1 Department of Orthopaedics, Tongji Hospital, Tongji Medical College, Huazhong University of Science and Technology, Wuhan 430030, China.; 2 Institute for Stem Cell Biology and Regenerative Medicine, Stanford University School of Medicine, Stanford, CA 94305, USA.; 3 Department of Surgery, Division of Plastic and Reconstructive Surgery, Stanford University School of Medicine, Stanford, CA 94305, USA.; 4 Department of Pathology, Tongji Hospital, Tongji Medical College, Huazhong University of Science and Technology, Wuhan 430030, China.

**Keywords:** Osteoarthritis, Cartilage degradation, BCLAF1, LAMTOR2, Signaling pathways

## Abstract

Osteoarthritis (OA) is a progressive degenerative joint disorder with cartilage degradation as the primary cause of joint pain and loss of joint function. B-cell lymphoma-2-associated transcription factor 1 (BCLAF1) is a key regulator of apoptosis and serves as a signal transducer of the NFκB and Hif-1α pathways, both of which are involved in osteoarthritic cartilage degradation. However, whether BCLAF1 contributes to the pathogenesis of OA remains unclear. The present study aims to elucidate the role of BCLAF1 in osteoarthritic cartilage degradation and the underlying mechanisms. We found that BCLAF1 levels were increased in cartilage tissue from OA patients, elder and surgery-induced OA mice, and primary chondrocytes treated with inflammatory cytokines. Knockdown of *Bclaf1* in chondrocytes inhibited the expression of catabolic factors and apoptosis rate, while promoting the expression of anabolic factors and enhancing chondrocyte functions such as viability and migration. Conversely, overexpression of *Bclaf1* produced the opposite effects. Furthermore, intra-articular injection of adenovirus containing shRNA targeting *Bclaf1* attenuated cartilage degradation and osteophytosis in a mouse OA model, while overexpression of BCLAF1 further aggravated cartilage degradation and osteophytosis *in vivo*. Through immunoprecipitation and protein mass spectrometry, we identified LAMTOR2 as a key mediator of BCLAF1 by regulating the translocation of BCLAF1 into the nucleus. Our findings reveal the critical role and key mechanisms of BCLAF1 in regulating cartilage degradation, representing a novel molecular target for the therapeutic development of OA.

## Introduction

Osteoarthritis (OA) is the most prevalent joint disease worldwide, characterized by progressive pain and irreversible loss of joint function 1, 2. Pathological changes in OA involve articular cartilage degradation, subchondral bone remodeling, and inflammation of the synovium and surrounding joint tissues 3. Accumulating evidence suggests that the degradation of articular cartilage in OA is actively regulated by chondrocytes rather than being a consequence of mechanical wear and tear 4, 5. In response to genetic, mechanical, and inflammatory factors, chondrocytes in OA undergo abnormal proliferation and apoptosis, as well as unbalanced anabolism and catabolism, leading to the breakdown of the cartilage matrix. Inflammatory cytokines such as tumor necrosis factor α (TNF-α), interleukin 1β (IL-1β), and interleukin 6 (IL-6) significantly contribute to OA pathogenesis by regulating chondrocytes 6, 7. Although animal studies have shown promising results, clinical trials targeting inflammatory cytokines have not been successful 8, 9, highlighting the complexity of OA pathogenesis and the urgent need for novel therapeutic targets.

B-cell lymphoma-2-associated transcription factor 1 (BCLAF1) is a multifaceted protein known for its involvement in apoptosis, transcriptional regulation, and DNA damage response 10. Although most studies suggest that BCLAF1 is an inducer of apoptotic and autophagic cell death in response to hypoxia 11, 12 and DNA damage 13, 14, a recent report shows that BCLAF1 also protects cells from TNF-induced apoptosis and tissue injury 15. Under hypoxic conditions, BCLAF1 is upregulated and is involved in the stabilization of hypoxia-inducible factor 1-alpha (Hif-1α) 11, 12. Upon exposure to irradiation or chemotherapeutic agents, BCLAF1 serves as an activated nuclear factor kappa B (NF-κB) signaling transducer in DNA damage-induced senescence 14. In cartilage, the Hif-1α signal is responsible for maintaining the homeostasis of chondrocytes under hypoxic conditions 16, while the NF-κB signal is critical in regulating chondrocyte catabolism in response to inflammatory cytokines 17. Given the regulatory roles of BCLAF1 in apoptosis and key chondrocyte-regulating signals, this protein may influence the pathophysiological processes underlying OA.

In the present study, we explored the role of BCLAF1 in osteoarthritis, hypothesizing that BCLAF1 contributes to OA pathogenesis through its involvement in chondrocyte apoptosis and inflammatory responses. By investigating the expression patterns and functional impacts of BCLAF1 in OA models, we demonstrated its potential as a biomarker and therapeutic target in the treatment of osteoarthritis. Additionally, we identified the specific mechanisms by which BCLAF1 influences OA progression, providing insight into the complex pathogenesis of OA.

## Materials and Methods

### Reagents and antibodies

Lipofectamine 3000 reagent was purchased from Invitrogen (Carlsbad, CA, USA). Recombinant mouse IL-1β was obtained from R&D Systems (Minneapolis, MN, United States). Anti-IκBα, p-IκBα, p65, p-p65, IκB kinase (IKK)- β, p-IKKα/β, p38, p-p38, c-Jun N-terminal kinase (JNK), p-JNK, extracellular signal-regulated kinase (ERK), p-ERK, Caspase3 and LAMTOR2 antibodies were supplied by Cell Signaling Technology (Beverly, MA, United States). Anti-BCLAF1, MMP3, MMP13, ACAN, COL2A1 and Cleaved Caspase3 antibodies were acquired from Abcam (Cambridge, United Kingdom). Anti-BAX and BCL-2 antibodies were bought from Proteintech Group (Rosemont, USA). Anti-GAPDH antibody was from Abclonal (Wuhan, China). The secondary antibodies were purchased from Jackson Lab (West Grove, PA, USA).

### Isolation and culture of primary chondrocytes

Cartilages of knee joints were isolated from 5-day-old newborn C57BL/6 mice to harvest the primary chondrocytes. After being digested by 0.2% trypsin (Boster Biotechnology, Wuhan, China) for 30 minutes, the cartilage pieces were purified by 0.25% collagenase II (Sigma-Aldrich, St. Louis, MO, USA) in the hybridization oven at 37 °C for 6 hours. The obtained chondrocytes were resuspended and cultured in the complete medium consisting of Dulbecco's Modified Eagle Medium (DMEM)/F12 medium (Hyclone, United States), 10% fetal bovine serum (FBS) (Gibco, Waltham, United States) and 100 units/mL penicillin-streptomycin (Boster Biotechnology, Wuhan, China) in a CO_2_ incubator, which is a humidified atmosphere of 5% CO_2_ at 37 °C. Chondrocytes were plated onto 10 cm plates and then used for experiments when grown to about 80% confluences.

### Small interfering RNA and plasmid transfections

Lipofectamine 3000 reagent (Invitrogen) was used for the transfection of *Bclaf1* and *Lamtor2* small interfering RNA (siRNA) or control siRNA (RiboBio, Guangzhou, China). In six-well plates, 50 pM *Bclaf1/Lamtor2* or control siRNA (si-*Bclaf1/Lamtor2* or si-Control), 5 μL Lipofectamine 3000, 250 µL Opti-MEM, and 2 mL complete medium were mixed and put into each well. The sequence of *Bclaf1* siRNA is 5'-CGTCGAGATTACAGGAATA-3' and *Lamtor2* siRNA is 5'-GCAGGTCAGGGTTAAGAGA-3'. The control ones consisted of scrambled siRNAs. As for the overexpression of *Bclaf1* or *Lamtor2*, chondrocytes were transfected with 1 µg *Bclaf1*/*Lamtor2* or control overexpression plasmids (p-*Bclaf1/Lamtor2* or p-Control) (OriGene, Rockville, MD, United States), mixed with 5 µL Lipofectamine 3000, 5 µL P3000 and 250 mL Opti-MEM in 2 mL complete culture medium. After 48 hours of transfection, chondrocytes were harvested for further experiments. The efficiency of knockdown and overexpression was confirmed by qPCR and western blotting.

### RNA extraction and RT-qPCR

Total RNA from cultured chondrocytes was extracted using TRIzol reagent (Takara Bio, Japan) according to the manufacturer's instructions. 1 μg RNA was used to synthesize complementary DNA (cDNA) using Hifair^®^ Ⅲ 1st Strand cDNA Synthesis SuperMix for qPCR (gDNA digester plus) (Yeasen Biotechnology, Shanghai, China). And real-time quantitative polymerase chain reaction (RT-qPCR) was performed using Hieff^®^ qPCR SYBR Green Master Mix (No Rox) (Yeasen Biotechnology, Shanghai, China). The sequences of all primers used are listed in **Table 1**. The relative mRNA levels of each gene were calculated by the 2^-ΔΔ^CT method and normalized to *Gapdh* 18.

### Protein extraction and western blotting

Total protein from cultured chondrocytes was extracted using radioimmunoprecipitation assay (RIPA) lysis buffer (Boster Biotechnology, Wuhan, China) mixed with 1% protease and phosphatase inhibitor (Boster Biotechnology, Wuhan, China). Equal amounts of protein were separated by 10-12% sodium dodecyl sulfate-polyacrylamide gel (SDS-PAGE) electrophoresis and then transferred to polyvinylidene fluoride (PVDF) membranes. The PVDF membranes were blocked with 5% non-fat milk or BSA in Tris-buffered saline with 0.1% Tween 20 buffer (TBST) for 1 hour at room temperature and incubated with the relevant primary antibodies on a shaker overnight at 4 °C. After incubation with corresponding horseradish peroxidase (HRP)-conjugated secondary antibodies for 1 h, proteins were visualized by Super ECL Detection Reagent (Yeasen Biotechnology, Shanghai, China) in the ChemiDoc XRS System (Bio-Rad Laboratories, Hercules, CA, United States), whose levels were quantified using Image lab software and normalized to GAPDH or relative total proteins.

### Flow cytometry

The apoptosis rate was determined using Annexin V-FITC/PI Apoptosis Detection Kit (Yeasen Biotechnology, Shanghai, China) by flow cytometry. Chondrocytes were collected and suspended in the binding buffer in a concentration of 1 × 10^5^ cells/mL, and stained with 5 µL Annexin V-FITC and 10 µL PI for 15 minutes in the darkroom. Then the patterns were analyzed using a flow cytometer (Becton Dickinson, United States), and the results were processed by FlowJo software (Treestar, Inc., San Carlos, CA, USA).

### Cell proliferation analysis

Primary chondrocytes plated onto 96-well plates were transfected with BCLAF1 siRNA or over-expressed plasmid and then stimulated with IL-1β for 48 hours. Cell proliferation was analyzed using Cell Counting Kit-8 Assay (CCK-8, TargetMol, United States) according to the provided instructions and all tests were performed in triplicate. After incubation in the CCK-8 culture medium for 1 hour, the samples were placed in a microplate reader (Bio Tek, United States) for the absorbance measurement at 450 nm.

### Cell migration assay

A two-well silicone culture insert (Ibidi, Gräfelfing, Germany) was set onto the middle of the well of 24-well plates, and 70 µL suspension with 5 × 10^5^ chondrocytes were seeded into each well of the chamber. After cell attachment, the silicone insert was removed, leaving a 500 mm cell-free gap. Then abundant complete culture medium was added to the well to fulfill the bottom. Images were captured at indicated time points using a Nikon inverted microscope (Nikon, Tokyo, Japan).

### Alcian blue staining and quantification

The prepared chondrocytes were digested, centrifuged and resuspended as 5 × 10^5^/20 µL, and inoculated 20 µL into the center of the 24-well plate to form chondrocyte micromass. After cultivation for 2 weeks with medium changed every 2 days, the cells were stained with 2% alcian blue liquor for 30 minutes and then washed with deionized water 3 times. After the pictures were scanned and captured with a microscope (Nikon, Tokyo, Japan), the stained micromass was covered with approximately 8 M guanidinium chloride dissolved in 2 M hydrochloric acid and shaken horizontally for a week. The absorbance at 630 nm of the solution was detected by a microplate reader (Bio Tek, United States).

### Co-immunoprecipitation

The interaction of BCLAF1 with LAMTOR2 proteins was measured in mice primary chondrocytes. After transfection of *Bclaf1-Flag* or *Lamtor2-Flag* plasmid with or without IL-1β stimulation, the chondrocytes were washed with PBS 3 times, lysed in NP-40 (Boster Biotechnology, Wuhan, China) mixed with 1% protease and phosphatase inhibitor and then collected in EP tubes to centrifuge for 30 minutes at 12000 rpm. The cleaned protein A/G magnetic beads (MCE, New Jersey, USA) were incubated with the indicated antibodies for 2 hours at 4 ℃. Next, the supernatant cell lysates were put into the beads-antibodies mixer and incubated together overnight at 4 ℃. After being washed 4 times, the immunoprecipitates were subjected to protein mass spectrometry or boiled in the loading buffer for western blotting.

### Animal model

Animals were purchased from the Experimental Animal Center of Tongji Hospital, Huazhong University of Science and Technology (Wuhan, China). All the animal experiments were approved by the Ethics Committee and the Institutional Animal Research Committee of Tongji Hospital (No. TJH-202001011). Destabilization of medial meniscus (DMM) surgery was used in our study to induce the animal OA model 19. 12-week-old male C57BL/6 mice were randomly equally divided into four groups: (1) Sham + Ad-shControl group (or Ad-Control or Control (saline with 1% DMSO)): sham-operated mice injected with Ad-shControl adenovirus (or Ad-Control adenovirus or Control) (n=6); (2) Sham + Ad-sh*Bclaf1* group (or Ad-*Bclaf1* adenovirus or Z-VAD-FMK(ZVF, MCE, New Jersey, USA)): sham-operated mice administrated with Ad-sh*Bclaf1* adenovirus (or Ad-*Bclaf1* adenovirus or ZVF) (n=6); (3) DMM + Ad-shControl group: DMM-operated mice treated with Ad-shControl adenovirus (or Ad-Control adenovirus or Control) (n=6); (4) DMM + Ad-sh*Bcalf1* group: DMM-operated mice injected with Ad-sh*Bclaf1* adenovirus (or Ad-*Bclaf1* adenovirus or ZVF) (n=6). The DMM surgery was performed as previously described 20. As for the sham ones, only the skin and joint capsule were opened, while other structures were kept intact. The adenovirus was designed and synthesized by Vigene Biosciences Inc. (Shandong, China). To knockdown or overexpress BCLAF1 or be a control *in vivo*, 10 µL adenovirus (1×10^9^ plaque-forming units (PFUs)/mL) was injected into each right knee joint gap 1 week after surgery (sham or DMM) once a week for 8 weeks using a 33G needle (Hamilton, Bonaduz, GR, Switzerland). The caspase inhibitor ZVF (100 μM in 1% DMSO, 10 µL) was injected into each right knee joint twice a week for 8 weeks to inhibit caspase *in vivo*. After injection for 8 weeks, all the mice were sacrificed and the knees were dissected for further experiments.

### Micro-computed tomography

After removing soft tissues, the right knees of mice were fixed in 4% paraformaldehyde for 48 h. Then the knees were scanned using high-resolution microcomputed tomography (micro-CT, Scanco Medical, Bassersdorf, Switzerland) at 15 mm resolution, 70 kVP, and 112 mA x-ray energy. The three-dimensional (3D) image reconstruction of the knees was performed according to the manufacturer's instructions. Quantitative analysis of the osteophyte score was performed according to previous studies 21, 22.

### Histopathological assessment

After micro-CT scanning, the knees were decalcified in 10% EDTA (pH 7.4) for 4 weeks. Samples were then embedded in paraffin and sectioned continuously at 5 µm thickness for toluidine blue, and Safranin O/Fast Green staining. The degree of articular cartilage damage was evaluated using the Osteoarthritis Research Association (OARSI) scoring system in a blind manner 23.

### Human samples

OA cartilages were obtained from patients who suffered OA and underwent total knee arthroplasty in Tongji Hospital. Normal cartilages were acquired from patients who did not have knee diseases or symptoms, but had to be amputated because of car accidents or some other diseases. The informed consent of the patients involved was obtained. All the collection and experiments involved human samples were approved by the Ethics Committee of the Tongji Hospital, Huazhong University of Science and Technology (No. TJ-IRB20210127).

### Immunohistochemistry analysis

The samples were sectioned into 5 mm slices, deparaffinized, hydrated, and blocked with BSA for 1 hour at room temperature. Subsequently, the sections were incubated with primary antibodies against BCLAF1 (1:100 dilution, Abcam), MMP3 (1:100 dilution, Abcam), MMP13 (1:100 dilution, Abcam), COL2A1 (1:200 dilution, Abcam), ACAN (1:100 dilution, Proteintech) overnight at 4 °C. Finally, the sections were incubated with corresponding secondary antibodies and counterstained with hematoxylin before being taken images under a microscope.

### Immunofluorescence analysis

Primary chondrocytes were seeded in the confocal dishes (Biosharp, Guangzhou, China) at a density of 1 × 10^5^/dish and cultured for 24 hours, followed by transfection of plasmids of *Bclaf1* and *Lamtor2* and pre-treatment with IL-1β. After being fixed in 4% paraformaldehyde for 15 minutes, permeabilized in 0.3% Triton X-100 for 5 minutes, and blocked with 5% BSA (dissolved in PBST) for 1 hour at room temperature, the chondrocytes were then incubated with anti-BCLAF1 and anti-LAMTOR2 antibodies overnight at 4℃ and secondary antibodies for 1 hour. The cell nucleus was stained with DAPI for 5 minutes. Images were captured with a laser confocal microscope (Olympus FV1000, Japan) in the experimental medical center of Tongji Hospital.

### Statistical analysis

All data were presented as means ± standard deviation (SD). Statistical analysis was performed using GraphPad Prism software (version 9.0, GraphPad Software Inc., San Diego, CA, United States). All experiments were repeated independently at least three times. The normality and equal variance of data were tested by the Shapiro-Wilk test and the F test respectively. Student's t-test was used to evaluate the statistical significance between two groups while one-way ANOVA followed by Turkey's test was used for multiple comparisons. P value < 0.05 was considered statistically significant.

## Results

### Increased BCLAF1 expression in articular cartilage of OA patients, aged and DMM mice and inflammatory factor-treated primary chondrocytes

**Figure 1:**
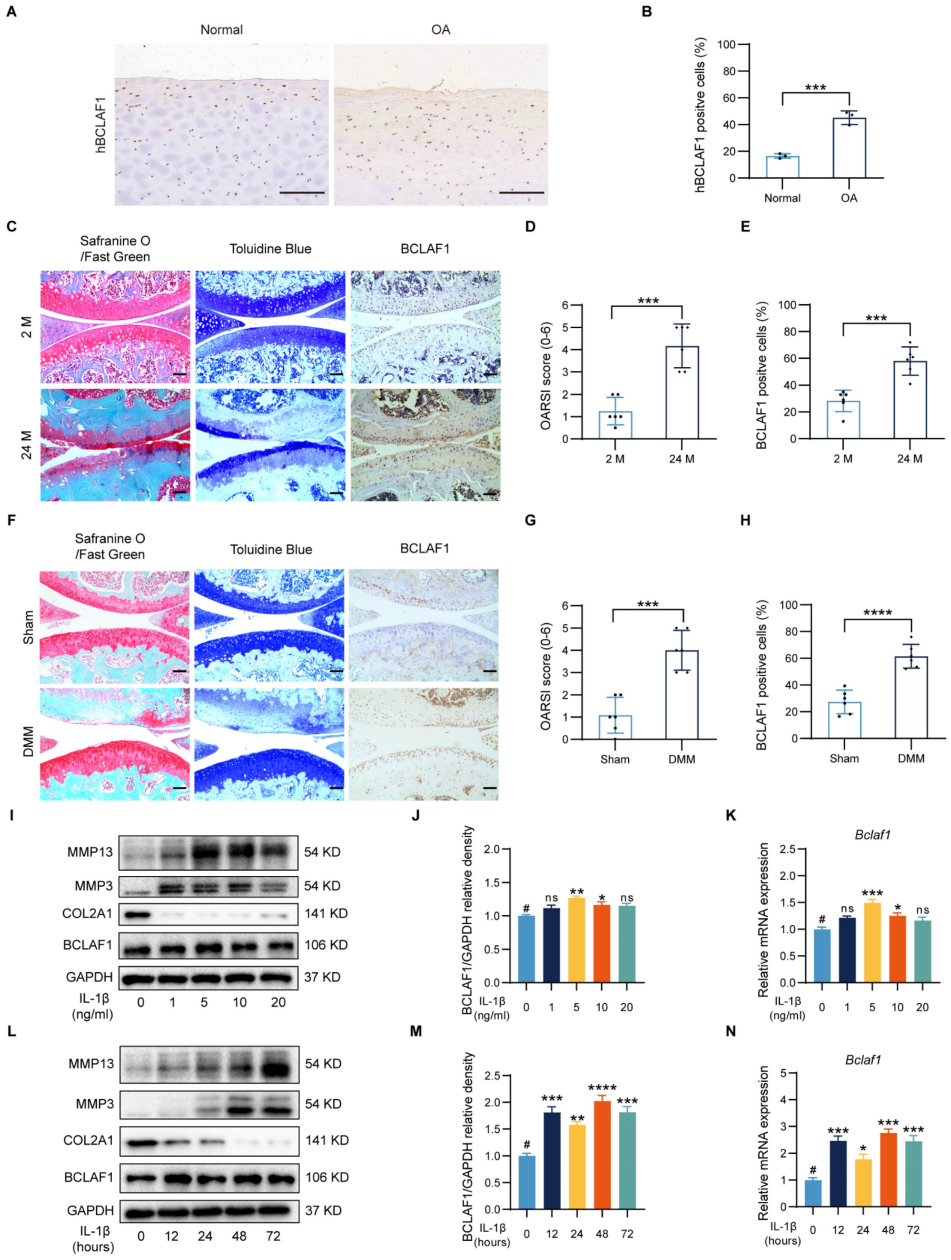
** Increased expression of BCLAF1 in articular cartilage of OA patients, aged and DMM mice, and IL-1β treated primary chondrocytes.** (**A**) Immunohistochemical staining of human BCLAF1 (hBCLAF1) of normal or OA knee cartilages from patients and (**B**) the quantification of the ratio of hBCLAF1 positive cells. Scale bar = 200 µm. (**C**) Safranin O/Fast Green staining (left), Toluidine Blue staining (middle), and immunohistochemical staining of BCLAF1 (right) of sagittal sections of knee joints from mice collected at 2 and 24 months of age. Scale bar = 100 µm. (**D**) OARSI grades of 2 and 24-month-old mice. (**E**) Quantification of immunohistochemical staining of BCLAF1 in articular cartilage. (**F**) Safranin O/Fast Green staining (left), Toluidine Blue staining (middle), and immunohistochemical staining of BCLAF1 (right) of sagittal sections of knee joints from DMM and sham-operated mice. Scale bar = 100 µm. (**G**) OARSI grades of DMM and sham-operated mice. (**H**) Quantification of immunohistochemical staining of BCLAF1 in articular cartilage. (**I**) Immunoblotting analysis of MMP13, MMP3, COL2A1, BCLAF1, and (**J**) Quantification of BCLAF1/GAPDH relative density. (**K**) mRNA level of *Bclaf1* determined by RT-qPCR in chondrocytes after stimulation with different concentrations of IL-1β (0, 1, 5, 10, and 20 ng/mL) for 48 h. (**L**) Immunoblotting analysis of MMP13, MMP3, COL2A1, BCLAF1, and (**M**) Quantification of BCLAF1/GAPDH relative density. (**N**) mRNA level of *Bclaf1* determined by RT-qPCR in chondrocytes after stimulation with 5ng/mL IL-1β for different periods (0, 12, 24, 48, and 72 h). All data are shown as mean ± SD, ns: no significant difference, ^*^p<0.05, ^**^p<0.01, ^***^p<0.001, ^****^p<0.0001 versus ^#^group.

It is fairly well-known that OA is strongly related to aging, and the degradation of cartilage gets worse with aging 24, 25. The cartilages from OA patients expressed more human BCALF1 (hBCLAF1) than the normal ones (**Figure 1A, B**). As presented in **Figure 1C**, compared to the young mice (2 months old, 2 M), the aged mice (24 months old, 24 M) showed obvious OA phenotypes in Safranine O/Fast green and Toluidine Blue staining with a significantly higher OARSI score (**Figure 1D**), which was consistent with previous cognition 24. Destabilization of medial meniscus (DMM) surgery was widely used to establish OA mouse models and induced significant OA phenotypes and higher OARSI scores compared to the sham groups (**Figure 1F, G**). To investigate the effects of BCLAF1 in OA, an immunocytochemical analysis was made and the results indicated that the expression of BCLAF1 elevated in the aged and DMM groups (**Figure 1E, H**). Due to the non-response of BCLAF1 to the stimulation of TNF-α (**Figure S1**), the primary chondrocytes were treated with different concentrations of IL-1β (0, 1, 5, 10, and 20 ng/mL) to mimic the OA inflammation *in vitro* and to explore the optimal stimuli conditions. The levels of MMP13 and MMP3 increased after IL-1β treatment in a dose-dependent way, while COL2A1 expression decreased in the meantime (**Figure 1I**). The expression of BCLAF1 rose both in mRNA and protein levels in this inflammatory environment and reached the peak at 5 ng/mL IL-1β treatment (**Figure 1I-K**). Therefore, 5 ng/mL IL-1β was used to treat chondrocytes for different time points (0, 12, 24, 48, and 72 hours). MMP13 and MMP3 levels increased gradually as time was prolonged, while the COL2A1 level was presented in an opposite manner (**Figure 1L**). Additionally, the expression of BCLAF1 was raised by IL-1β and reached the peak at 48 hours, however there was no obvious trend with time (**Figure 1L-N**). Accordingly, stimulation of 5 ng/mL IL-1β for 48 hours was used in the following experiments. Our results demonstrated that BCLAF1 was up-regulated in the OA process *in vivo* and *in vitro*.

### BCLAF1 promotes inflammatory factor-induced chondrocyte catabolism

**Figure 2:**
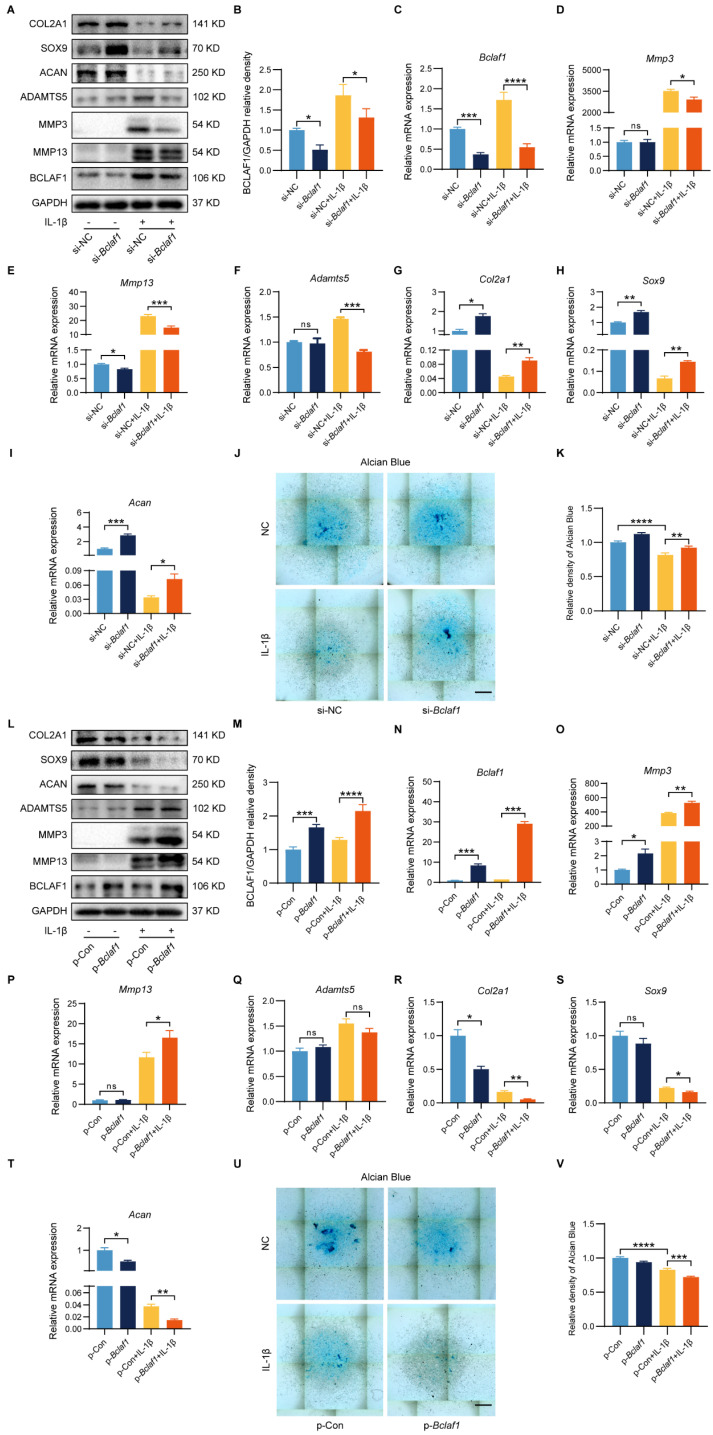
** BCLAF1 regulates IL-1β induced mouse cartilage degradation of osteoarthritis *in vitro*.** Primary chondrocytes were transfected with si-NC or si-*Bclaf1* for 48 h and then treated with or without IL-1β (5 ng/mL) for 48 h. (**A**) Immunoblotting analysis of BCLAF1, MMP13, MMP3, ADAMTS5, ACAN, SOX9 and COL2A1. (**B**) Quantitative analysis of BCLAF1/GAPDH relative density. (**C-I**) mRNA levels of *Bclaf1*, catabolic factors (*Mmp3*, *Mmp13*, *Adamts5*) and anabolic factors (*Col2a*, *Sox9*, *Acan*). (**J**) Representative images of Alcian Blue staining of each group and (**K**) their quantitative analysis of absorbance at 630 nm. Primary chondrocytes were transfected with p-Con or p-*Bclaf1* for 48 h and then treated with or without IL-1β (5 ng/mL) for 48 h (**L**) Immunoblotting analysis BCLAF1, MMP13, MMP3, ADAMTS5, ACAN, SOX9 and COL2A1. (**M**) Quantitative analysis of BCLAF1/GAPDH relative density. (**N-T**) mRNA levels of *Bclaf1*, catabolic factors (*Mmp3*, *Mmp13*, *Adamts5*) and anabolic factors (*Col2a*, *Sox9*, *Acan*). (**U**) Representative images of Alcian Blue staining of each group and (**V**) their quantitative analysis of absorbance at 630 nm. Scale bar = 500 µm. All data are shown as mean ± SD, ns: no significant difference, ^*^p<0.05, ^**^p<0.01, ^***^p<0.001, ^****^p<0.0001.

To elucidate the functional role of BCLAF1 in inflammatory factor-induced chondrocyte catabolism *in vitro*, siRNA or plasmid was used to knockdown or overexpress BCLAF1 in primary mouse chondrocytes. The efficiency of BCLAF1 knockdown and overexpression was verified by RT-qPCR and western blotting (**Figure 2A-C and L-N**). Moreover, the knockdown of BCLAF1 lessened the expressions of catabolic factors such as MMP13, MMP3, and ADAMTS5 at both protein and mRNA levels raised by IL-1β (**Figure 2A, D-F**). The protein and mRNA levels of anabolic factors such as ACAN, SOX9, and COL2A1 increased after BCLAF1 knockdown (**Figure 2A, G-I**). Knockdown of BCLAF1 could also induce the generation of alcian blue which was hampered by IL-1β (**Figure 2J, K**). In addition, the overexpression of BCLAF1 notably increased the elevation of MMP13 and MMP3 induced by IL-1β at protein and mRNA levels (**Figure 2L, O, P**). However, the alteration of both ADAMTS5 protein and mRNA levels was not significant (**Figure 2L, Q**). Consistently, the overexpression of BCLAF1 further reduced the protein and mRNA levels of ACAN, SOX9, and COL2A1 decreased by IL-1β (**Figure 2L, R-T**) and further degenerated alcian blue caused by IL-1β (**Figure 2U, V**). To sum up, the data above showed that BCLAF1 promoted inflammatory factor-induced chondrocyte catabolism *in vitro*.

### BCLAF1 suppresses the viability and migration of chondrocytes

**Figure 3:**
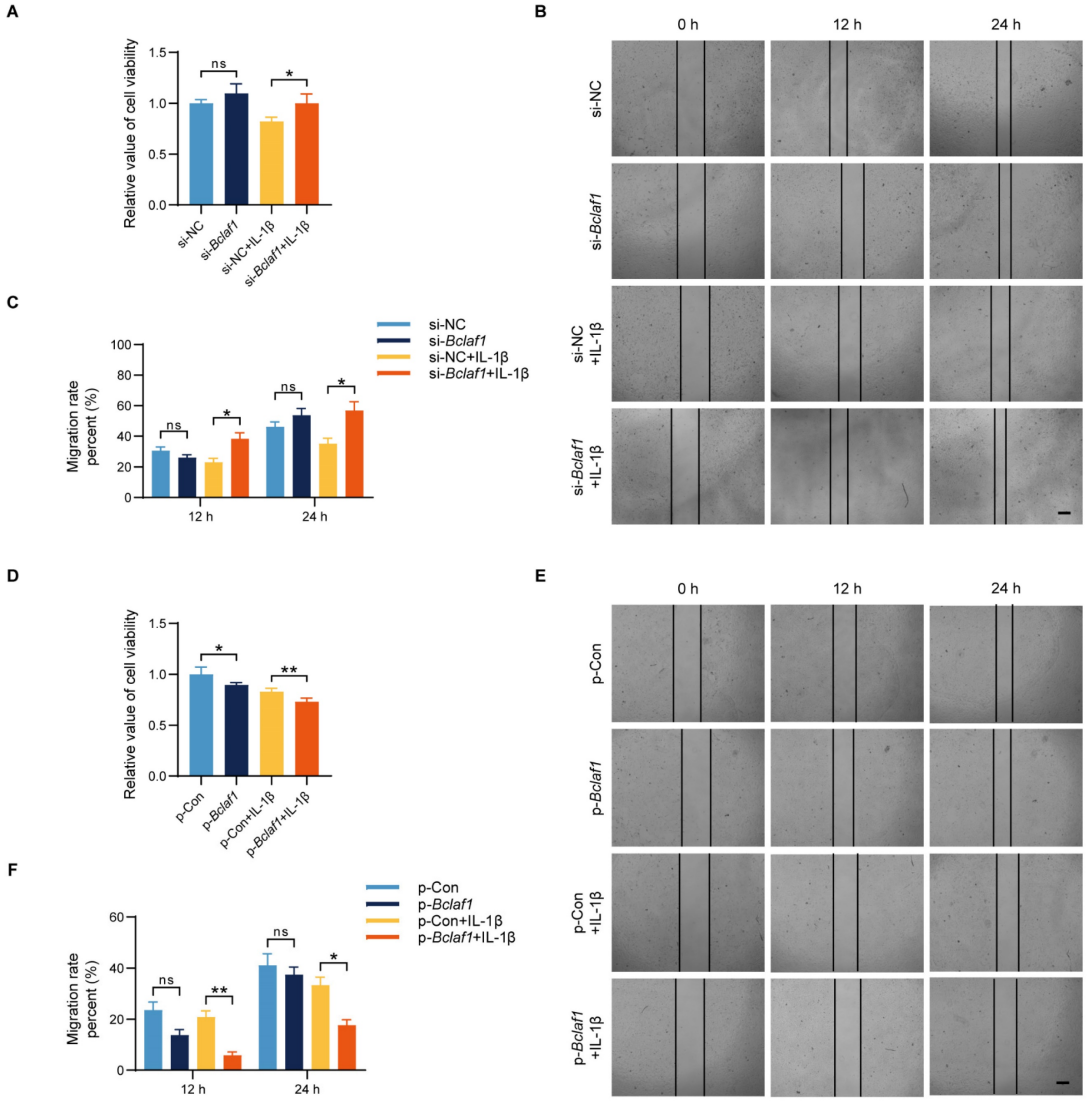
** BCLAF1 suppresses the viability and migration of chondrocytes.** (**A**) Effect of knockdown of BCLAF1 on the viability of primary chondrocytes with or without IL-1β treatment (5 ng/mL) for 48 h, detected by CCK8 assay. (**B**) The scratch-wound assay and (**C**) quantitative data revealed the impacts of transfection with BCLAF1 knock-down for different time points (0, 12, 24 h) on the migration of chondrocytes. (**D**) Effect of overexpression of BCLAF1 on the viability of primary chondrocytes with or without IL-1β treatment (5 ng/mL) for 48 h, detected by CCK8 assay. (**E**) The scratch-wound assay and (**F**) quantitative data revealed the impacts of transfection with BCLAF1 overexpression for different time points (0, 12, 24 h) on the migration of chondrocytes. Scale bar = 100 μm. All data are shown as mean ± SD, ns: no significant difference, ^*^p<0.05, ^**^p<0.01.

To investigate the effect of BCLAF1 on cell functions such as cell viability and migration of chondrocytes, we performed cell proliferation assays and cell migration assays. Results showed that knockdown of BCLAF1 increased the cell viability when cells were stimulated with IL-1β at the same time, while overexpression of BCLAF1 did the opposite (**Figure 3A, D**). Additionally, cell migration was promoted by knockdown of BCLAF1 in the presence of IL-1β stimulation at the time points of 12 h and 24 h (**Figure 3B, C**). In contrast, the overexpression of BCLAF1 suppressed cell migration with or without IL-1β stimulation (**Figure 3E, F**). Above all, BCLAF1 depressed cell viability and migration.

### BCLAF1 promotes inflammatory factor-induced chondrocyte apoptosis

**Figure 4:**
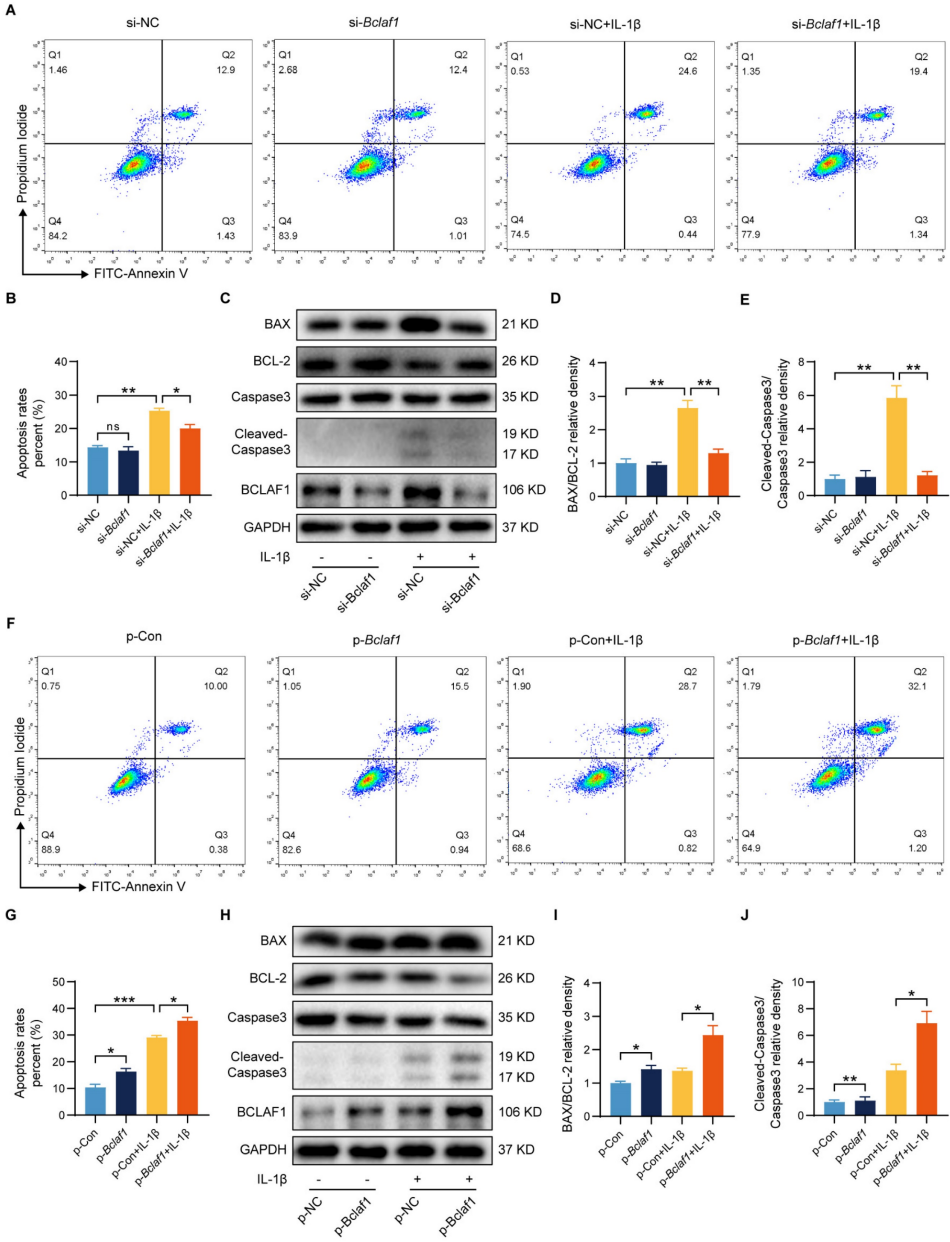
** BCLAF1 promotes cartilage degeneration induced by IL-1β by regulation of cell apoptosis.** (**A**) Apoptosis of primary chondrocytes was detected by flow cytometry of Annexin V-FITC/PI staining after si-NC or si-*Bclaf1* transfection with or without IL-1β treatment (5 ng/mL) for 48 h and (**B**) quantification data of the apoptosis rates (Q2+Q3). (**C**) Immunoblotting analysis of BAX, BCL-2, Caspase 3, and Cleaved-Caspase 3. (**D**) Quantification data of BAX/BCL-2 and (**E**) Cleaved-Caspase 3/Caspase 3 relative density. (**F**) Apoptosis of primary chondrocytes was detected by flow cytometry of Annexin V-FITC/PI staining after p-Con or p-*Bclaf1* transfection with or without IL-1β treatment (5 ng/mL) for 48 h and (**G**) quantification data of the apoptosis rates (Q2+Q3). (**H**) Immunoblotting analysis of BAX, BCL-2, Caspase 3, and Cleaved-Caspase 3. (**I**) Quantification data of BAX/BCL-2 and (**J**) Cleaved-Caspase 3/Caspase 3 relative density. All data are shown as mean ± SD, ns: no significant difference, ^*^p<0.05, ^**^p<0.01, ^***^p<0.001.

The dysregulation of cell apoptosis has been confirmed to be involved in OA pathophysiology 26, 27, and BCLAF1 is closely associated with apoptosis 15, 28. We thus performed flow cytometry and western blotting to explore whether BCLAF1 regulated IL-1β induced inflammation in chondrocytes by influence on cell apoptosis. As shown in **Figure 4A and B**, the knockdown of BCLAF1 reduced the apoptosis rate elevated by IL-1β. Whereas overexpression of BCLAF1 further raised the apoptosis rate induced by IL-1β (**Figure 4F, G**). The results of western blotting were consistent with that of flow cytometry. When chondrocytes were transfected with BCLAF1 siRNA, the expression of pro-apoptosis factors like BAX and Cleaved-Caspase3 decreased, while the expression of the anti-apoptosis factor like BCL-2 increased (**Figure 4C**). Furthermore, the ratio of BAX/BCL-2 and Cleaved-Caspase3/Caspase3, representing the promotion of apoptosis, declined (**Figure 4D, E**). And vice versa (**Figure 4H, I, J**). These results manifested that BCLAF1 promoted cell apoptosis of chondrocytes under inflammation stress induced by IL-1β.

### Caspase inhibitor rescues osteoarthritic cartilage degradation *in vivo*

Chondrocyte apoptosis is closely associated with osteoarthritis (OA) progression 26, 27. To investigate whether blocking caspase, a pivotal apoptosis mediator, can counteract osteoarthritic cartilage degradation, the caspase inhibitor Z-VAD-FMK (ZVF) was administered in an OA mouse model. DMM surgery was used to induce the OA mouse model. One week after surgery, either the control solution (saline with 1% DMSO) or ZVF was intra-articular injected twice weekly for eight weeks in both sham-operated and DMM groups. Histological assessments, including Safranin O/Fast Green, Toluidine Blue, and immunohistochemical staining, were conducted to evaluate OA severity. The efficiency of caspase inhibitor ZVF in the cartilage was confirmed by immuno-histochemical staining in the DMM mice (**Figure S2A, B**). ZVF treatment significantly mitigated OA phenotypes and lowered OARSI scores induced by DMM surgery (**Figure S2C, D**). Moreover, DMM groups exhibited elevated MMP13 expression, which was notably reduced following ZVF injection, whereas COL2A1 expression that diminished in DMM groups was restored upon caspase inhibition (**Figure S2E-G**). To assess the impact of caspase inhibition on osteophyte formation, knee joints were analyzed via micro-CT scans. Results revealed fewer osteophytes in the DMM + ZVF group compared to the DMM + Control group (**Figure S2H, I**). Collectively, these findings demonstrate that caspase inhibition can effectively prevent cartilage degradation and osteophyte formation in the OA mouse model.

### Knockdown of BCLAF1 ameliorates osteoarthritic cartilage degradation *in vivo*

**Figure 5:**
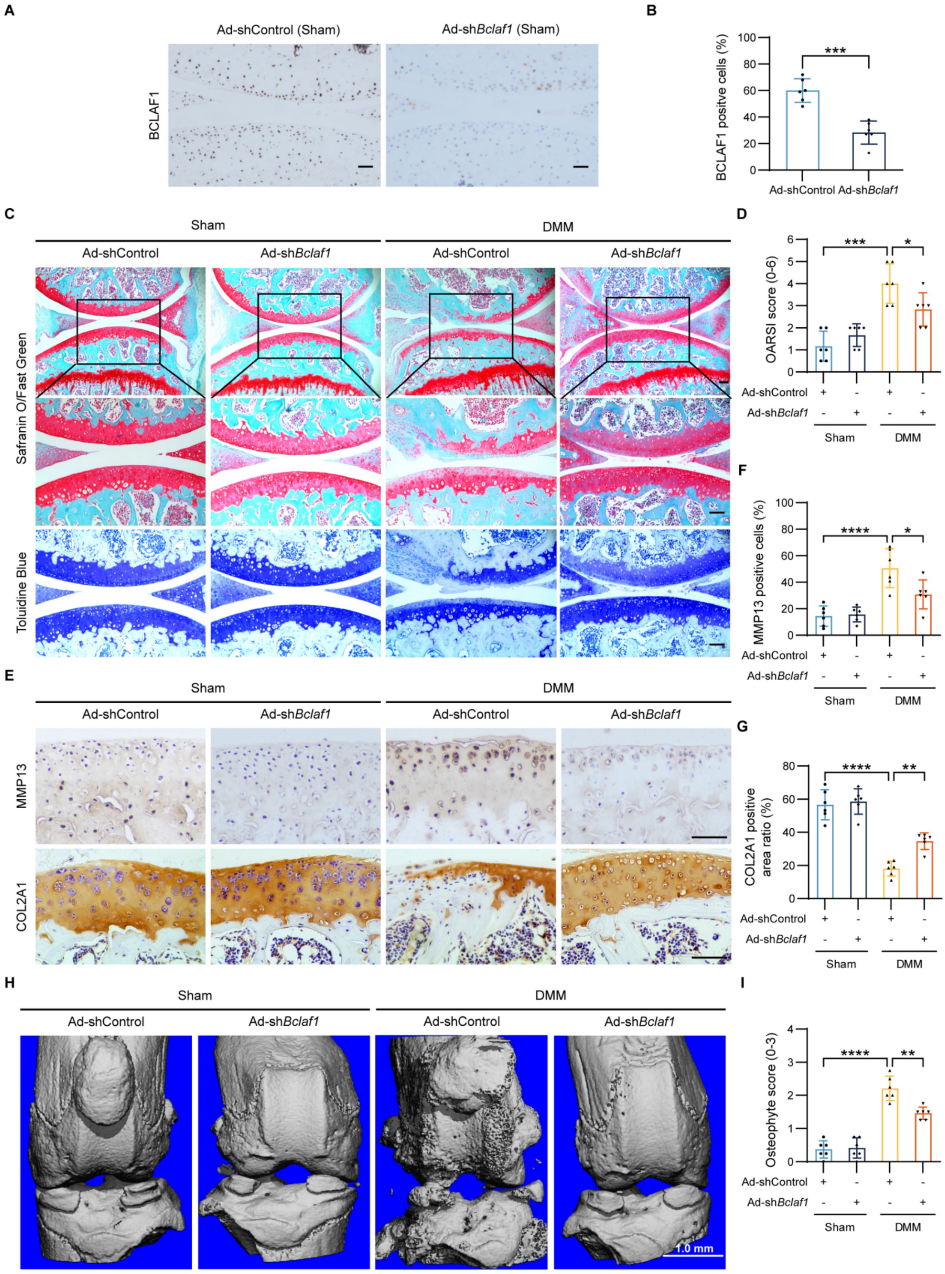
** Knockdown of BCLAF1 ameliorates osteoarthritic cartilage degradation *in vivo***. (**A**) Immunohistochemical staining of BCLAF1 of sagittal sections of knee joints of sham-operated mice and (**B**) its quantification. (**C**) Safranin O/Fast Green staining and Toluidine Blue staining and (**D**) their OARSI scores. (**E**) Immunohistochemical staining of MMP13 and COL2A1, and (**F, G**) their quantification of sagittal sections of knee joints in the indicated groups. All data are shown as mean ± SD, ^*^p<0.05, ^**^p<0.01, ^***^p<0.001, ^****^p<0.0001. Scale bar = 100 µm. (**H**) Representative micro-CT images of knee joints in indicated groups and (**I**) their osteophyte scores. Scale bar = 1.0 mm.

To explore the potential of BCLAF1 as a therapeutic target for OA, we tested the effect of BCLAF1 knockdown in the OA mouse model. One week after surgery, Ad-shControl or Ad-sh*Bclaf1* adenovirus was intra-articular injected in both sham-operated and DMM groups once a week for eight weeks. The efficiency of BCLAF1 knockdown in the cartilage was confirmed by immunohistochemical staining (**Figure 5A, B**). Compared to sham-operated groups, the DMM groups presented more severe OA phenotypes and higher OARSI grades, which however was alleviated by knock-down of BCLAF1 (**Figure 5C, D**). Besides, the expression of MMP13 was remarkably increased in DMM groups and was decreased by injection of Ad-sh*Bclaf1* compared with Ad-shControl. The expression of COL2A1, on the contrary, was significantly reduced in DMM groups and elevated by down-regulation of BCLAF1 (**Figure 5E-G**). Additionally, to investigate the impact of BCLAF1 on osteophyte formation, the knees were scanned with micro-CT. As shown in **Figure 5H and 5I**, fewer osteophytes were formed in the DMM + Ad-sh*Bclaf1* group than in the DMM+ Ad-shControl group. In summary, our results illustrated that the knockdown of BCLAF1 attenuates cartilage degradation and osteophyte formation in the OA mouse model.

### Overexpression of BCLAF1 aggravates osteoarthritic cartilage degradation *in vivo*

**Figure 6:**
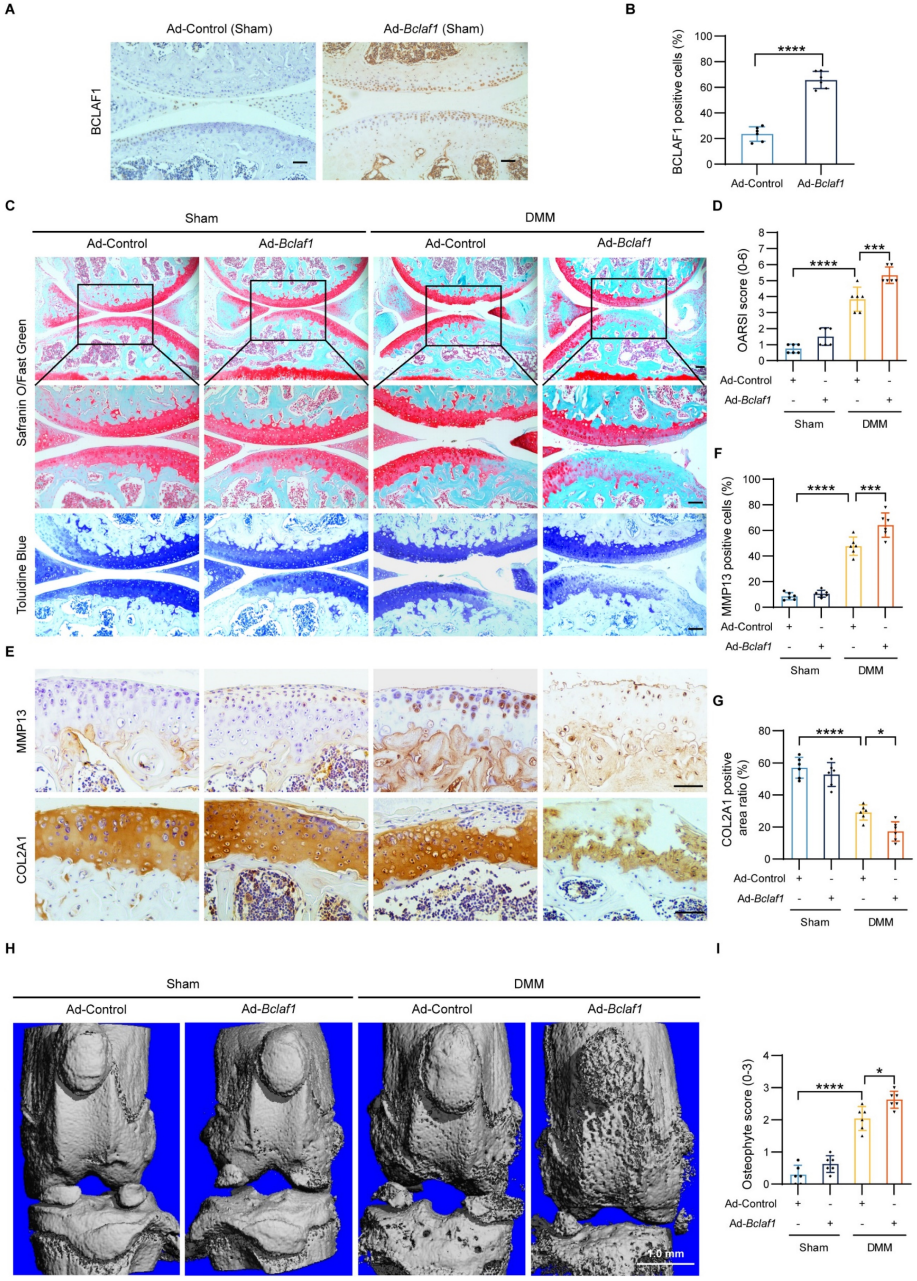
** Overexpression of BCLAF1 aggravates osteoarthritic cartilage degradation *in vivo***. (**A**) Immunohistochemical staining of BCLAF1 of sagittal sections of knee joints of sham-operated mice and (**B**) its quantification. (**C**) Safranin O/Fast Green staining and Toluidine Blue staining and (**D**) their OARSI scores. (**E**) Immunohistochemical staining of MMP13 and COL2A1, and (**F, G**) their quantification of sagittal sections of knee joints in the indicated groups. All data are shown as mean ± SD, ^*^p<0.05, ^**^p<0.01, ^***^p<0.001, ^****^p<0.0001. Scale bar = 100 µm. (**H**) Representative micro-CT images of knee joints in indicated groups and (**I**) their osteophyte scores. Scale bar = 1.0 mm.

To fully understand the pathophysiological role of BCLAF1 in OA, we further tested the effect of BCLAF1 overexpression in the OA mouse model. The methodology and process were similar to the above. Immunohistochemical staining validated successful BCLAF1 overexpression in cartilage (**Figure 6A, B**). DMM groups displayed pronounced OA phenotypes and higher OARSI scores compared to sham controls, which were further exacerbated by BCLAF1 overexpression (**Figure 6C, D**). MMP13 expression, already elevated in DMM groups, was significantly heightened with Ad-*Bclaf1* injection. In contrast, COL2A1 expression, reduced in DMM groups, was further suppressed by BCLAF1 overexpression (**Figure 6E-G**). Knee joints subjected to micro-CT scans revealed increased osteophyte formation in the DMM + Ad-*Bclaf1* group relative to the DMM + Ad-Control group (**Figure 6H, I**). These results consistently demonstrate that BCLAF1 overexpression accelerates cartilage degradation and osteophyte formation in the OA mouse model.

### BCLAF1 detaches from LAMTOR2 and translocates into nucleus under inflammatory factor treatment in chondrocytes

**Figure 7:**
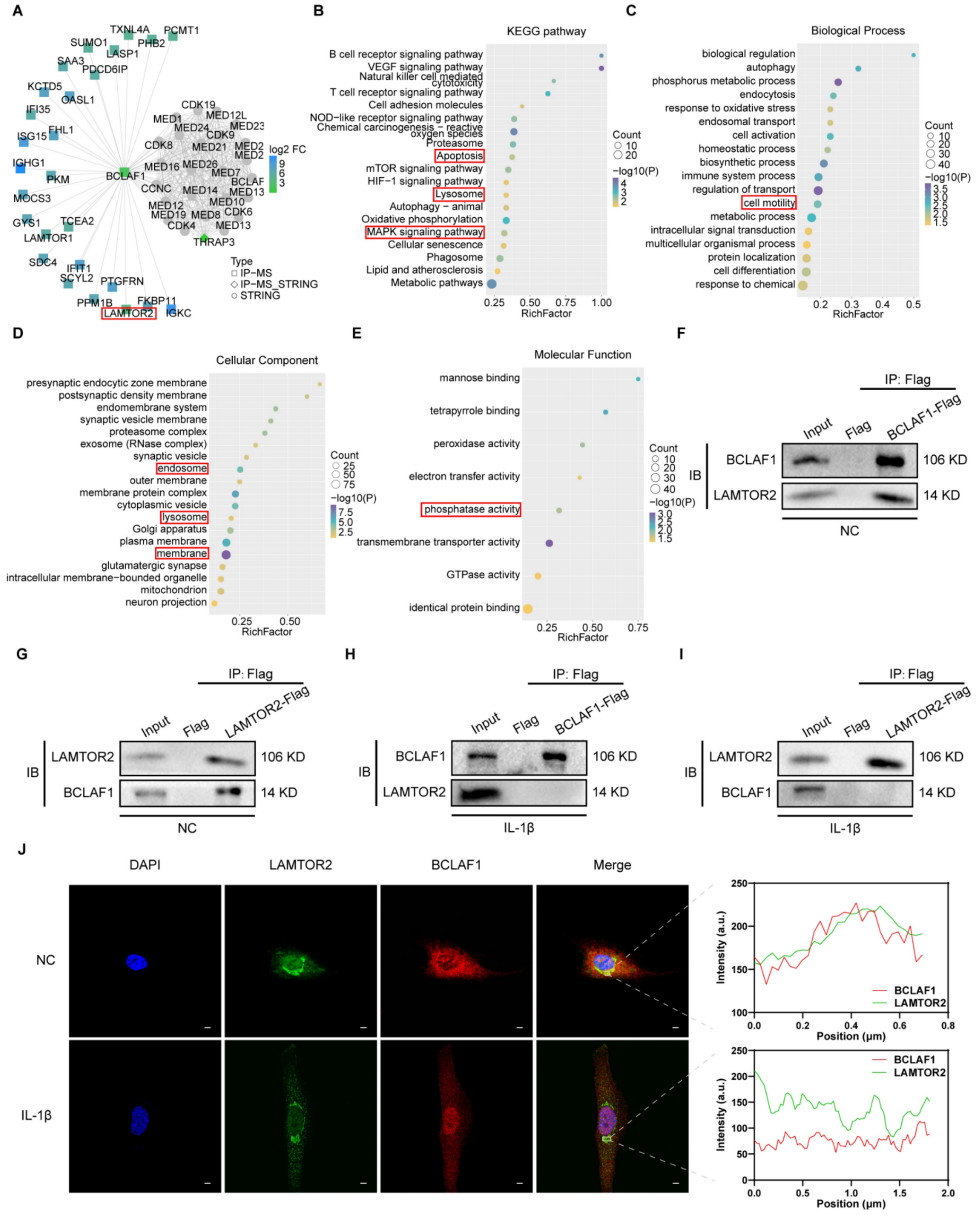
** BCLAF1 detaches from LAMTOR2 and translocates into nucleus under inflammatory factor treatment in chondrocytes.** (**A**) PPI analysis of immunoprecipitation of BCLAF1 followed by protein mass spectrometry shows the possible interactor of BCLAF1. (**B**) KEGG analysis (**C**) GO. Biological Process (**D**) GO. Cellular Components and (**E**) GO. Molecular Function shows the relationship of BCLAF1 in primary chondrocytes. (**F, G**) Co-immunoprecipitation followed by immunoblotting analysis was used to detect the interaction of BCLAF1 and LAMTOR2 in primary chondrocytes without and (**H, I**) with IL-1β treatment (5 ng/mL) for 48 h. (**J**) Left: Images captured from the confocal microscope of immunofluorescence staining of the nucleus (DAPI, blue), LAMTOR2 (green), BCLAF1 (red), and their merged images in primary chondrocytes with or without IL-1β treatment (5 ng/mL) for 48 h. Right: Fluorescence intensity analysis of BCLAF1 and LAMTOR2 in the white rectangle area. Scale bar = 5 μm.

The role of BCLAF1 in OA has been proved through our results above, but the mechanism remained elusive. To shed light on how BCLAF1 participated in OA, immunoprecipitation and protein mass spectrometry were performed. After screening the possible interactors in the PPI result, LAMTOR2 attracted our attention (**Figure 7A**). Ragulator complex protein LAMTOR2 (also named as late endosomal/lysosomal adaptor and MAPK and MTOR activator 2) could serve as a part of Ragulator complex implicated in activation of mTORC1 and MAPK 29-31, both of which were closely coupled with OA verified by previous studies 32-34. The KEGG and GO analysis indicated the potential pathways and some other factors associated with BCLAF1 (**Figure 7B-E**). The interaction of BCLAF1 and LAMTOR2 in primary chondrocytes was confirmed by co-immunoprecipitation (CO-IP) and IL-1β could break this interaction (**Figure 7F-I**). Additionally, BCLAF1 and LAMTOR2 were stained with immunofluorescence in chondrocytes to provide further insights into their interaction. Without IL-1β stimulation, BCLAF1 mainly localized in the cytoplasm, and LAMTOR2 concentrated near the nucleus (**Figure 7J**), consistent with previous studies that reported LAMTOR2 mainly existed in lysosome and endosome 29. The merged image and fluorescence intensity analysis showed a good colocalization of LAMTOR2 and BCLAF1 (**Figure 7J, right top panel**). However, when IL-1β treated, BCLAF1 was transported into the nucleus while LAMTOR2 remained in place, leading to the abrogation of their interaction (**Figure 7J, right bottom panel**). In conclusion, BCLAF1 interacted with LAMTOR2 near the nucleus in primary chondrocytes and detached to translocate into the nucleus under the stimulation of IL-1β.

### LAMTOR2 mediates the effect of BCLAF1 on chondrocytes catabolism

**Figure 8:**
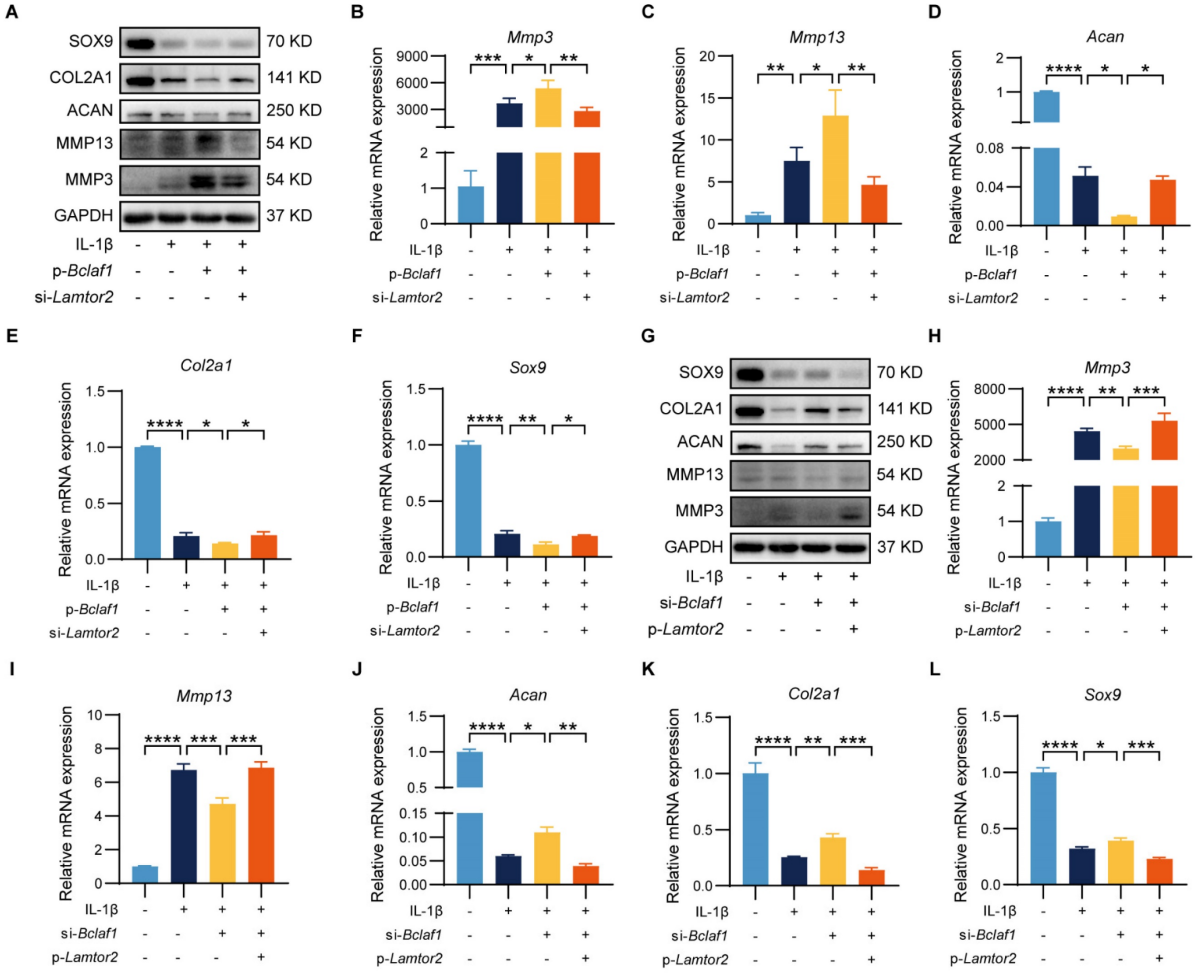
** LAMTOR2 mediates the effect of BCLAF1 on chondrocyte catabolism.** (**A**) Immunoblotting analysis and (**B-F**) mRNA levels of catabolic factors (*Mmp3*, *Mmp13*, *Adamts5*) and anabolic factors (*Acan*, *Col2a*, *Sox9*) after primary chondrocytes were transfected with p-*Bclaf1* and si-*Lamtor2* for 48 h and then treated with or without IL-1β (5 ng/mL) for 48 h. (**G**) Immunoblotting analysis and (**H-L**) mRNA levels of catabolic factors (*Mmp3*, *Mmp13*, *Adamts5*) and anabolic factors (*Acan*, *Col2a*, *Sox9*) after primary chondrocytes were transfected with si-*Bclaf1* and p-*Lamtor2* for 48 h and then treated with or without IL-1β (5 ng/mL) for 48 h. All data are shown as mean ± SD, ^*^p<0.05, ^**^p<0.01, ^***^p<0.001, ^****^p<0.0001.

Given that LAMTOR2 could interact with BCLAF1, which was regulated by IL-1β, we further tested whether BCLAF1 tapped into its effects on chondrocytes through LAMTOR2. To unravel this problem, primary chondrocytes were transfected with p-*Bclaf1* and si-*Lamtor2*, or p-*Lamtor2* and si-*Bclaf1* to see whether regulation of LAMTOR2 could counter the effects of BCLAF1 on chondrocytes. As the results showed, knock-down of LAMTOR2 via si-*Lamtor2* could decrease the expressions of MMP3 and MMP13 raised by overexpression of BCLAF1 via p-*Bclaf1*, and enhance the expressions of SOX9, COL2A1, and ACAN blunted by overexpression of BCLAF1 both at protein and RNA levels (**Figure 8A-F**). On the contrary, overexpression of LAMTOR2 via p-*Lamtor2* could also reverse the effects of knock-down of BCLAF1 via si-*Bclaf1* both at protein and RNA levels (**Figure 8G-L**). These data showed that LAMTOR2 mediated the effect of BCLAF1 on chondrocyte catabolism.

### BCLAF1 activates MAPK and NF-κB signaling pathways in chondrocytes

**Figure 9:**
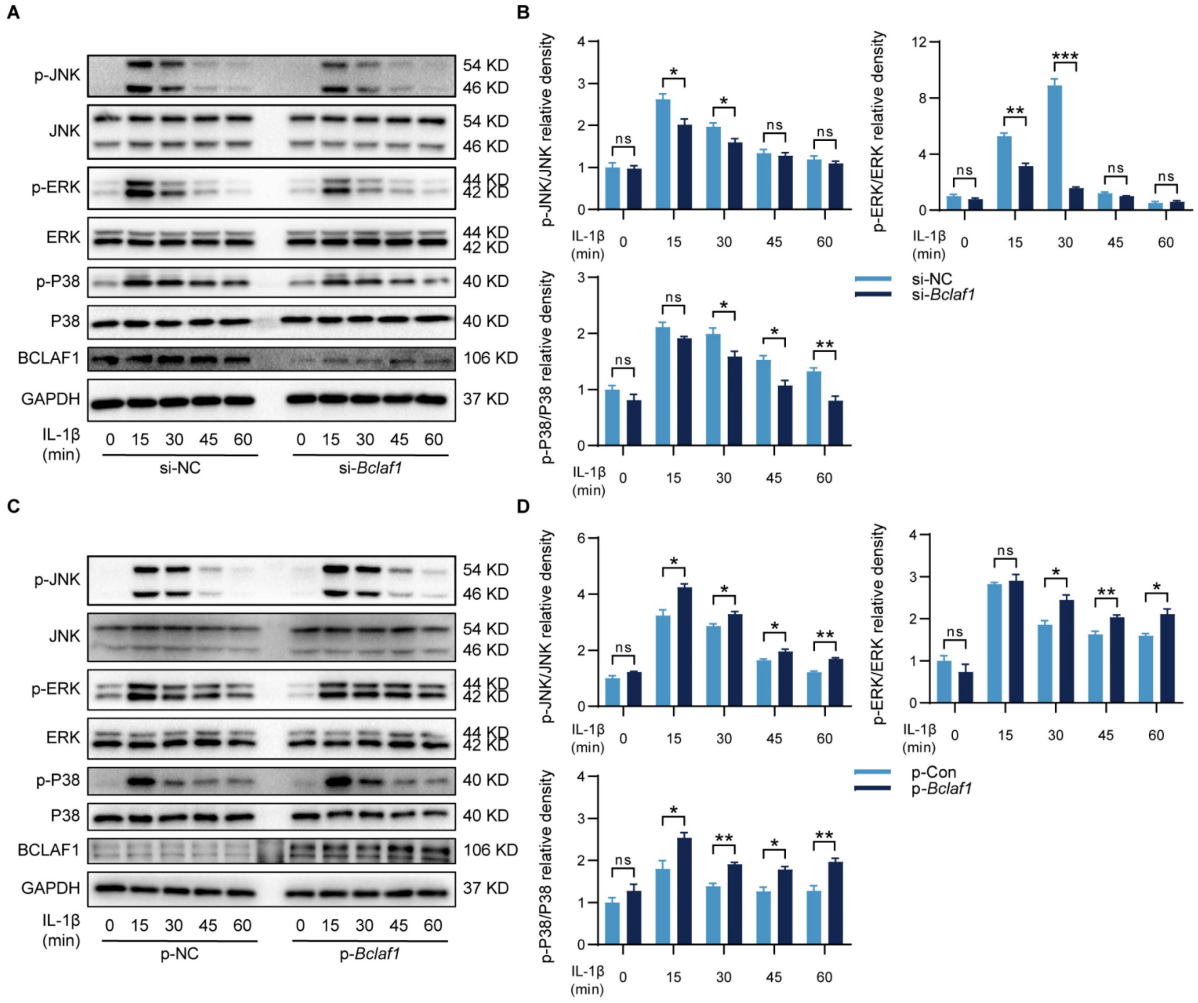
** BCLAF1 activates MAPK signaling pathway in chondrocytes.** Primary chondrocytes were transfected with siRNA or plasmid for 48 h followed by starvation with culture medium without serum, and then stimulated with IL-1β for indicated time points (0, 15, 30, 45, 60 min). Representative western blot of the effect of BCLAF1 (**A**) silencing and (**C**) overexpression on phosphorylated JNK, ERK, and P38 in chondrocytes. Quantification of the effect of BCLAF1 (**B**) silencing and (**D**) overexpression on the ratio of phosphorylated JNK, ERK, and P38 to their total protein respectively in chondrocytes. All data are shown as mean ± SD, ns: no significant difference, ^*^p<0.05, ^**^p<0.01, ^***^p<0.001.

KEGG analysis of protein mass spectrometry showed that the MAPK signaling pathway might be involved in the effects of BCLAF1 on OA (**Figure 7B**). Thus, the changes in phosphorylation levels of related components in the MAPK signaling pathway were detected to determine whether BCLAF1 affects the activation of the MAPK signaling pathway. As the results showed, the phosphorylated JNK, ERK, and P38 levels were reduced by knock-down of BCLAF1 (**Figure 9A and B**), and elevated by overexpression of BCLAF1 (**Figure 9C and D**).

**Figure 10:**
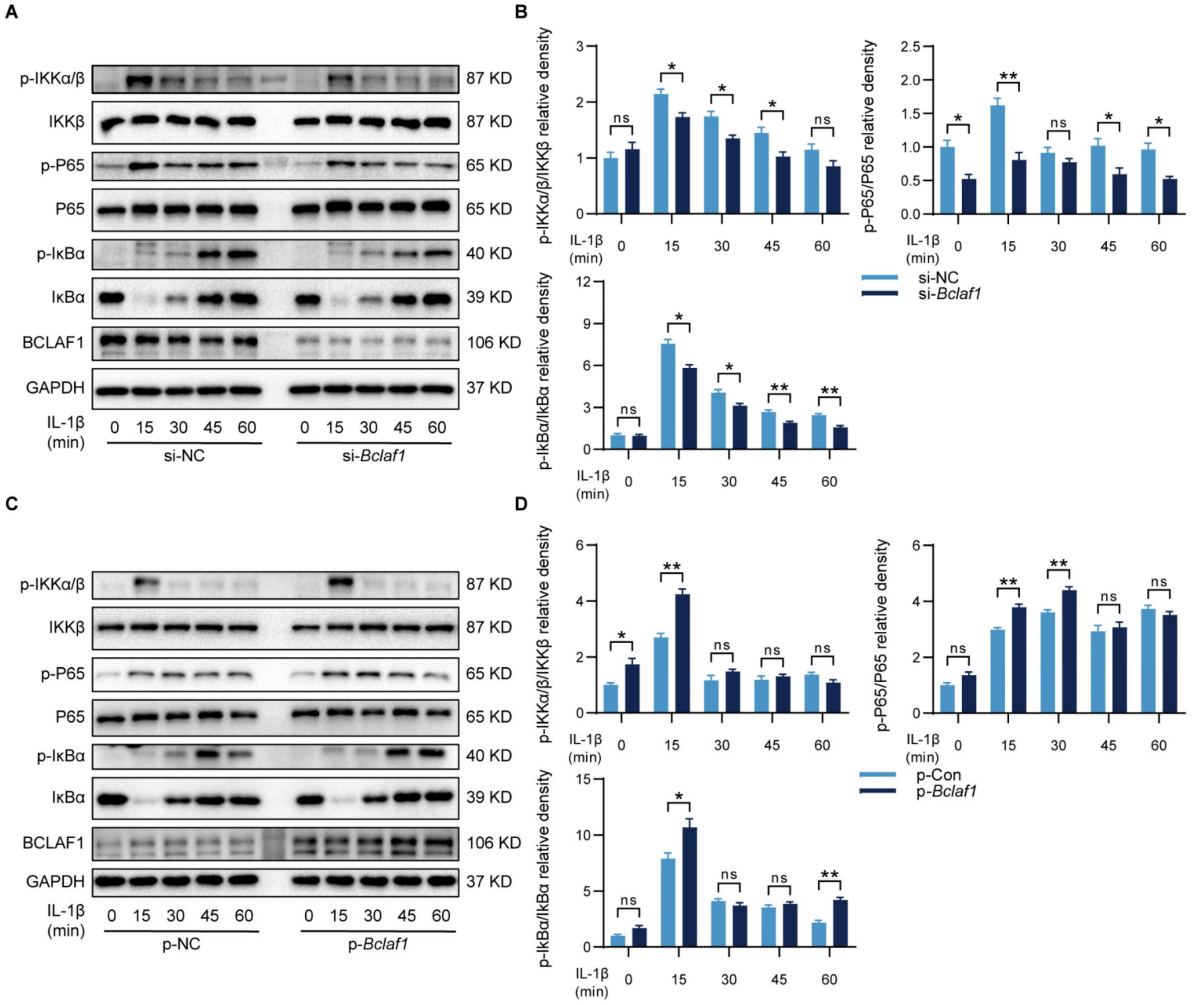
** BCLAF1 activates NF-κB signaling pathway in chondrocytes.** Primary chondrocytes were transfected with siRNA or plasmid for 48 h followed by starvation with culture medium without serum, and then stimulated with IL-1β for indicated time points (0, 15, 30, 45, 60 min). Representative western blot of the effect of BCLAF1 (**A**) silencing and (**C**) overexpression on phosphorylated IKKα/β, IκBα and P65 in chondrocytes. Quantification of the effect of BCLAF1 (**B**) silencing and (**D**) overexpression on the ratio of phosphorylated IKKα/β, IκBα, and P65 to their total protein respectively in chondrocytes. All data are shown as mean ± SD, ns: no significant difference, ^*^p<0.05, ^**^p<0.01.

In some other diseases, BCLAF1 was reported to play an important role in the NF-κB signaling pathway 14, which has also been known as a vital participator of OA 32, but the relationship of BCLAF1 and NF-κB signaling pathway in OA needs elucidating. Therefore, the influence of BCLAF1 on the NF-κB signaling pathway was also detected. As shown in **Figure 10**, the phosphorylation of IKKα/β, IκBα, and P65 was diminished by down-regulation of BCLAF1 and increased after BCLAF1 plasmid transfection. The results above outlined that BCLAF1 promoted cartilage degeneration of OA by activating MAPK and NF-κB signaling pathways.

## Discussion

OA is the most common form of arthritis and the dominant cause of pain and disability of the elderly over the world, which is characterized by degeneration of articular cartilage, changes of subchondral bone, osteophyte formation, synovial inflammation, degradation of ligaments and menisci, and hypertrophy of the joint capsule 2, 3, 35. Cartilage degeneration is considered the most important pathological change responsible for OA symptoms like pain and stiffness 4, 36. Articular cartilage is composed of chondrocytes and the extracellular matrix derived from them, besides water. The extracellular matrix, mainly type II collagen, aggrecan, and other proteoglycans, forms a network providing tensile strength and compressive resilience for the articular cartilage to be capable of loading 2, 37, 38. Under pathological circumstances, chondrocytes produce several cytokines such as IL-1β, MMPs, and ADAMTS, most of which can degrade the extracellular matrix and lead to cartilage destruction, contributing to the progression of OA 3, 39, 40. However, the underlying mechanism of cartilage degeneration of OA still remains elusive.

In our present study, the expression of BCLAF1 was increased in articular cartilage of aged and DMM mice, and IL-1β treated primary chondrocytes. Moreover, BCLAF1 knock-down decreased the expression of catabolic factors like MMPs and ADAMTS5, and induced alcian blue generation, while BCLAF1 overexpression brought about contrary results. Also, BCLAF1 could affect cell functions of chondrocytes under IL-1β treatment, that was BCLAF1 knock-down promoted chondrocytes viability and migration and BCALF1 overexpression did the opposite. Nevertheless, BCLAF1 knock-down suppressed chondrocyte apoptosis while BCLAF1 overexpression enhanced chondrocyte apoptosis. The interaction of BCLAF1 and LAMTOR2 played an indispensable role in OA. In a physiological situation, BCLAF1 existed mainly in the cytoplasm and interacted with LAMTOR2. When IL-1β treatment, however, BCLAF1 detached from LAMTOR2 and translocated into the nucleus. Regulation of LAMTOR2 could reverse the effect BCLAF1 had on chondrocytes. MAPK and NF-κB pathways were abrogated by BCLAF1 knock-down and activated by BCLAF1 overexpression. Additionally, BCLAF1 knock-down *in vivo* attenuated cartilage destruction, MMP13 expression, and osteophytes, but elevated COL2A1 levels in DMM mice. In summary, our findings suggest a definite role of BCLAF1 in cartilage degradation of OA.

BCL-2 family proteins, composed of both pro-apoptotic and anti-apoptotic members, are the key to cell apoptosis, which is one of the most important biological processes for life 41-43. The factors able to combine with and influence BCL-2 family proteins can also regulate apoptosis and take part in various physiological and pathological changes, e.g. BCLAF1 44, 45. Several tumors have been proven to be regulated by BCLAF1. For instance, recently Peipei Zhang, et al. found that the glycolysis and tumor progression of esophageal squamous cell carcinoma were promoted by BCLAF1 via YTHDF2-SIX1 pathway in a manner of m^6^A 46. Notably, abundant studies have demonstrated the close relationship between BCALF1 and hepatocellular carcinoma, showing that BCLAF1 could influence hepatocellular carcinoma by binding to SPOP 47, or targeting lncRNA NEAT1 48, or regulating c-MYC mRNA stability 49, or some other ways 12. Moreover, BCLAF1 also played a critical role in hypoxia. Under hypoxia, Hif-1α enhanced the transcription of BCLAF1, and BCLAF1 bound to Hif-1α and protected it from degradation in return 11. In this study, BCLAF1 expression was enhanced in the elder mice and IL-1β treated primary chondrocytes. To explore the optimal stimuli conditions due to the different sensitivity proteins may have in responding to inflammatory factors, the chondrocytes were stimulated with a gradient of IL-1β concentrations and durations, resulting in the increasing of BCLAF1 expression, peaking at 5 ng/mL and 48 hours, which was used as the optimal stimuli in the following experiments. However, no trend was observed with the changes of IL-1β concentration or duration, potentially owing to the imbalances in compensatory mechanisms and inflammatory responses. Similar patterns have been observed in other studies 50-53, suggesting that further researches are needed to explore the precise mechanisms underlying these phenomena. Regulation of BCLAF1 significantly affected the expression of catabolic and anabolic factors, and the cell functions such as vitality and migration of IL-1β treated chondrocytes. Knock-down of BCLAF1 *in vivo* alleviated cartilage degradation and osteophytes in DMM mice.

Apoptosis, a kind of programmed cell death required for living organisms, has been reported to take charge of the modulation of OA 26, 54. F Héraud, et al. found the chondrocytes apoptosis rate was increased from 2-5% to 18-21% in OA cartilage compared to normal cartilage, indicating the important role of apoptosis in the evolution of OA 27. J-H Ryu, et al. revealed that HIF-2α fostered chondrocytes apoptosis by enhancing *Fas* expression and amplifying downstream signaling, leading to OA cartilage destruction 55. Another research demonstrated that chondrocyte apoptosis was affected by OSCAR (osteoclast-associated receptor) deficiency through blunting TRAIL (tumor necrosis factor-related apoptosis-inducing ligand) expression, and contributed to OA pathogenesis 56. Our study showed that BCLAF1 knock-down inhibited the expression of BAX and Cleaved-Caspase 3, while increasing the expression of BCL-2, and suppressed chondrocytes apoptosis induced by IL-1β. The results of BCLAF1 overexpression were quite the opposite. Moreover, inhibiting caspase in the cartilage could rescue osteoarthritic cartilage degradation *in vivo*. These findings indicated that BCLAF1 promoted OA by enhancing chondrocyte apoptosis.

LAMTOR2 is a composition of Ragulator/LAMTOR complex famous for modulation of mTOR and MAPK pathways and the key to stabilization and cytosolic localization of the rest complex components 29. Previous studies showed that LAMTOR2 was involved in several biological processes like homeostasis of Langerhans cells and dendritic cells 57, 58, cell migration 59, and pancreatic ductal adenocarcinoma 60. In this study, LAMTOR2 was found binding to BCLAF1 in cytoplasm under normal circumstances. However, IL-1β broke this interaction and led to the nuclear translocation of BCLAF1. Interestingly, translocation of BCALF1 has been proven to contribute to cardiac ischemia-reperfusion injury 61. It is worth noting that our analysis results of protein mass spectrometry showed a close relationship between BCLAF1 and chondrocytes apoptosis and cell motility, consistent with our previous findings. Endosome, lysosome, and membrane were also enriched in the analysis, where LAMTOR2 was exactly located. Importantly, we found that regulation of LAMTOR2 could reverse the effects of BCLAF1 on cartilage degeneration of OA. Therefore, our findings clarified the certain role of LAMTOR2 in BCLAF1 regulating OA.

MAPK and NF-κB pathways are coupled with OA cartilage degradation 32-34. MAPK pathway was enriched in our KEGG analysis. The correlation between BCLAF1 and NF-κB pathways has been demonstrated 14. Remarkably, phosphatase activity was enriched in our GO analysis. Accordingly, we investigated the role of BCALF1 in the activation of MAPK and NF-κB pathways in the OA process. The results showed that silencing BCLAF1 hampered IL-1β-induced phosphorylation of JNK, ERK, and P38 in the MAPK pathway, and IKKα/β, IκBα, and P65 in the NF-κB pathway. Inversely, overexpressing BCLAF1 further augmented IL-1β induced phosphorylation of the factors above. Our findings suggested that BCLAF1 promoted cartilage degeneration of OA by activation of MAPK and NF-κB pathways.

**Figure 11:**
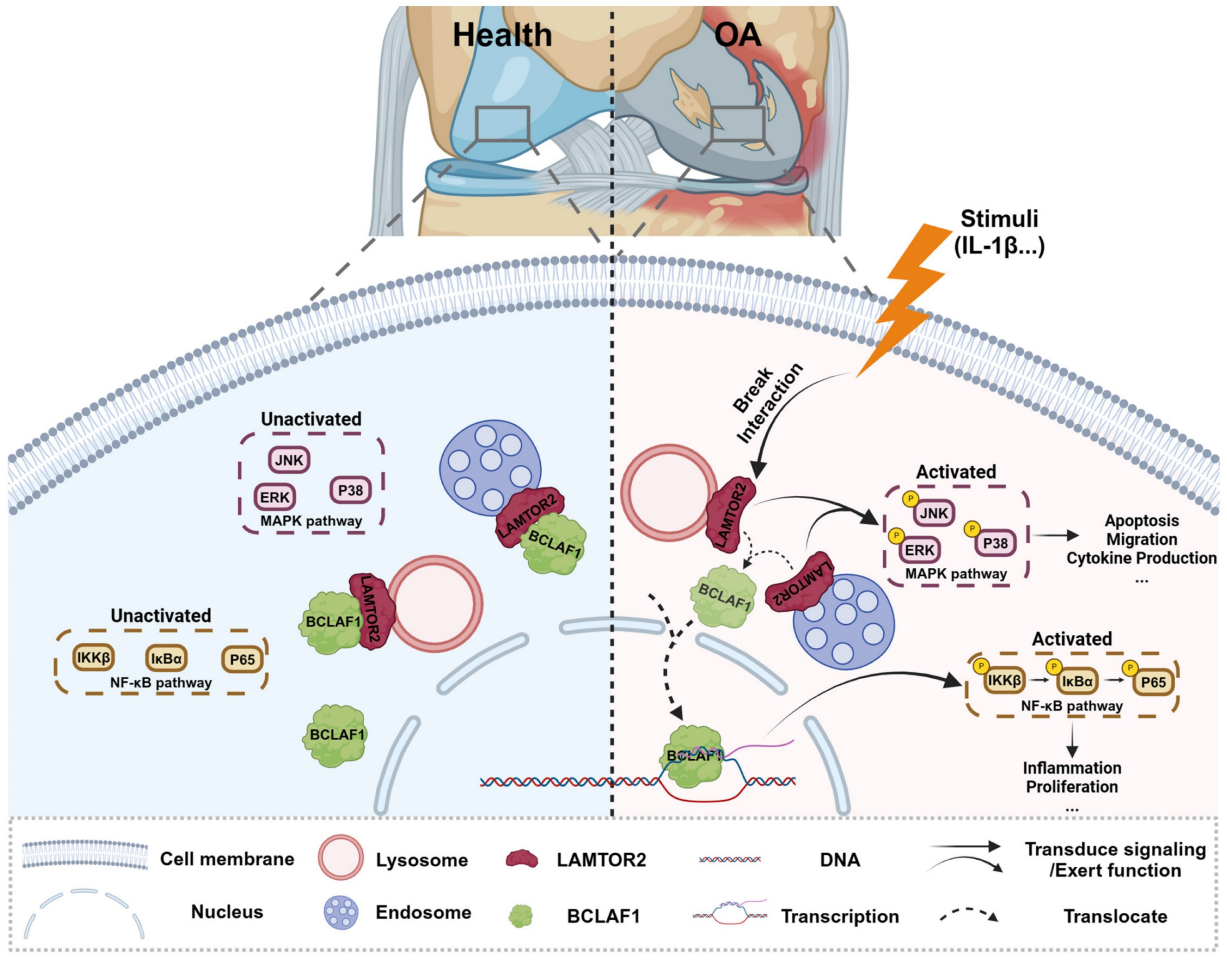
** Schematic representation of BCLAF1 promotes osteoarthritic cartilage degradation by division from LAMTOR2.** BCLAF1 interacts with LAMTOR2 near the nucleus and departs to translocate into the nucleus upon IL-1β treatment, and activates MAPK and NF-κB pathways to promote chondrocytes catabolism and cartilage degradation during the OA process. Created with Biorender.com.

Collectively, our present study showed a critical role of BCLAF1 in cartilage degradation of OA. BCLAF1 bound to LAMTOR2 in the cytoplasm and detached to translocation into the nucleus to activate NF-κB and MAPK pathways, both of which promoted cartilage degeneration of OA in turn. Accordingly, we hypothesized that LAMTOR2 may function mainly as an interactor with BCLAF1 and limit its nuclear translocation, only when BCLAF1 departed from LAMTOR2 and translocated to the nucleus, MAPK, and NF-κB pathways were subsequently activated, resulting in OA phenotypes (**Figure 11**). Further investigation is required for more comprehensive and precise mechanisms.

## Supplementary Material

Supplementary figures.

## Figures and Tables

**Table 1:** Sequence of primers used in this study

Primers	Sense (from 5' to 3')	Antisense (from 5' to 3')
*Bclaf1*	GGCTGCTTGCTAGTACACTTGTCC	TGCTGGCCTGTGGCAACTTAATG
*Gapdh*	CTCCCACTCTTCCACCTTCG	GCTGTAGCCGTATTCATT
*Mmp3*	ACTCCCTGGGACTCTACCAC	GGTACCACGAGGACATCAGG
*Mmp13*	TGATGGACCTTCTGGTCTTCTGG	CATCCACATGGTTGGGAAGTTCT
*Sox9*	CAGCCCCTTCAACCTTCCTC	TGATGGTCAGCGTAGTCGTATT
*Acan*	AGGTGTCGCTCCCCAACTAT	CTTCACAGCGGTAGATCCCAG
*Col2a1*	CTTCACAGCGGTAGATCCCAG	ACCAGGGGAACCACTCTCAC
*Adamts5*	CCCAGGATAAAACCAGGCAG	CGGCCAAGGGTTGTAAATGG
*Lamtor2*	GCTTTGACGCAGGTGCTAAG	TGTCCCCATAACCGGAGTAGG

## Abbreviations

ACAN: aggrecan
ADAMTS: a disintegrin and metalloproteinase with thrombospondin motifs
BCLAF1: B-cell lymphoma-2-associated transcription factor 1
COL2A1: collagen type II alpha 1
DMM: destabilization of medial meniscus
h: hours
HE: hematoxylin and eosin
Hif-1α: hypoxia-inducible factor 1-alpha
IL-1β: interleukin 1β
IL-6: interleukin 6
LAMTOR2: late endosomal/lysosomal adaptor and MAPK and MTOR activator 2
MAPK: mitogen-activated protein kinase
MMPs: matrix metallopeptidases
NF-κB: nuclear factor kappa B
OA: osteoarthritis
OARSI: Osteoarthritis Research Association
SD: standard deviation
TNF-α: tumor necrosis factor α
