# Supplementary material for: BCLAF1 Regulates Osteoarthritic Cartilage Degradation Through Interaction with LAMTOR2
Source: Int J Biol Sci. 2025 Feb 3;21(4):1666–85. doi: 10.7150/ijbs.100396 (PMC11844276; doi:10.7150/ijbs.100396)
Supplement: Supplementary file 1 — Supplementary figures. [file ijbsv21p1666s1.pdf]

Supplementary Materials

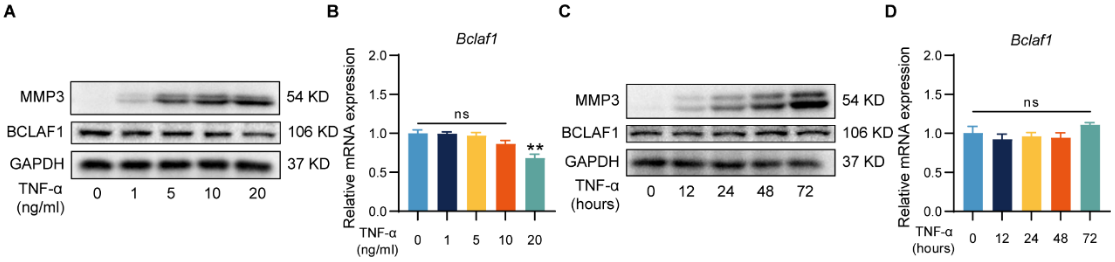

Figure S1. BCLAF1 does not respond to TNF-α stimulation in primary chondrocyts. (**A**) Immunoblotting analysis of MMP3 and BCLAF1, and (**B**) mRNA level of *Bclaf1* determined by RT-qPCR in chondrocytes after stimulation with different concentrations of TNF-α (0, 1, 5, 10, and 20 ng/ml) for 48 h. (**C**) Immunoblotting analysis of MMP3 and BCLAF1, and (**D**) mRNA level of *Bclaf1* determined by RT-qPCR in chondrocytes after stimulation with 5ng/ml TNF-α for different periods (0, 12, 24, 48, and 72 h). All data are shown as mean ± SD, ns: no significant difference, **p<0.01 versus the control group (0 ng/ml or 0 h TNF-α treatment).

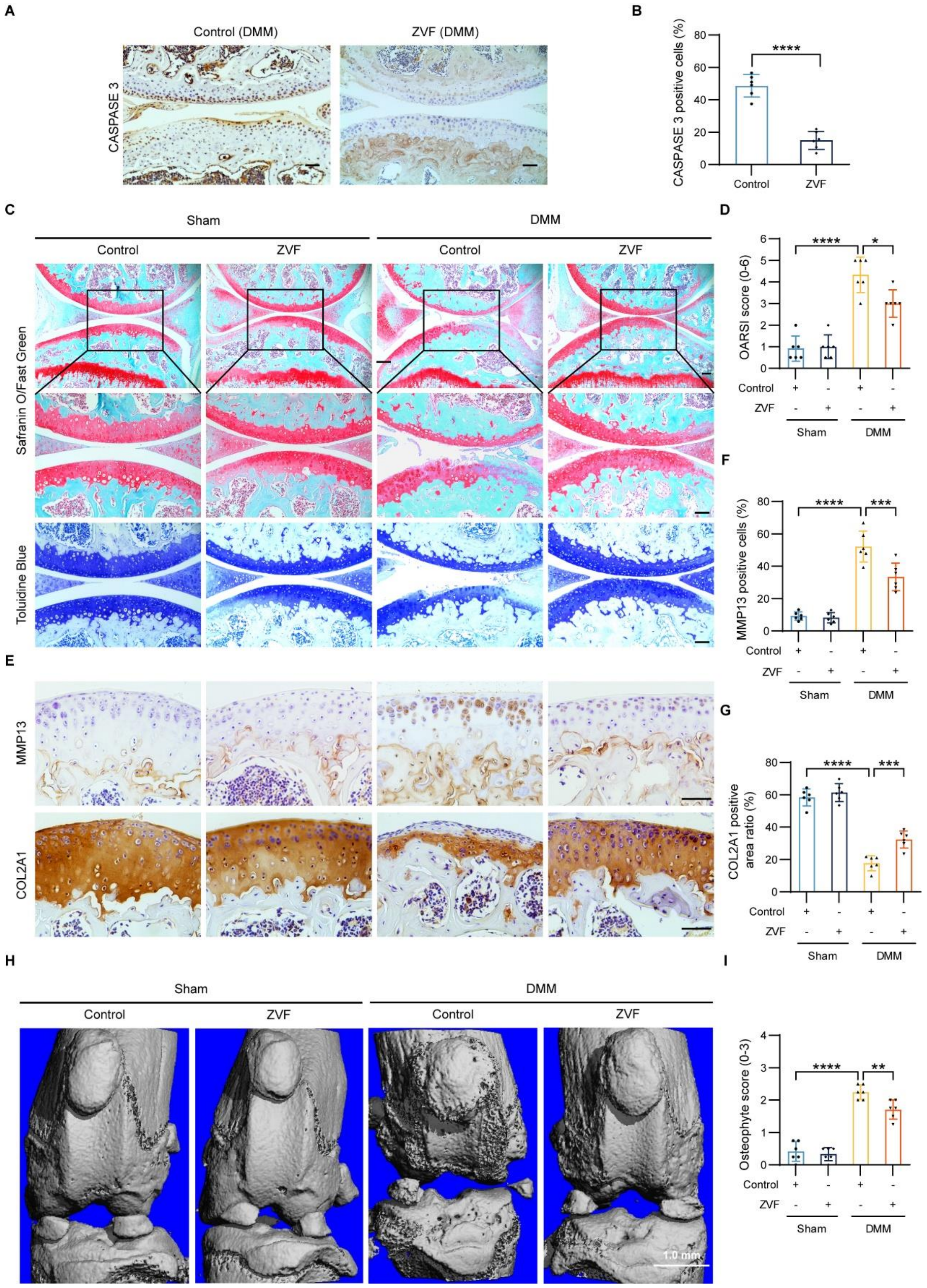

Figure S2. Caspase inhibitor rescues osteoarthritic cartilage degradation *in vivo*. (**A**) Immunohistochemical staining of BCLAF1 of sagittal sections of knee joints and (**B**) its quantification. (**C**) Safranin O/Fast Green staining and Toluidine Blue staining and (**D**) their OARSI scores. (**E**) Immunohistochemical staining of MMP13 and COL2A1, and (**F, G**) their quantification of sagittal sections of knee joints in the indicated groups. All data are shown as mean ± SD, $^{*}p<0.05$, $^{**}p<0.01$, $^{***}p<0.001$,

$^{****}p<0.0001$. Scale bar = 100 µm. (**H**) Representative micro-CT images of knee joints in indicated groups and (**I**) their osteophyte scores. Scale bar = 1.0 mm.
